# The *Plasmodium falciparum* STEVOR Multigene Family Mediates Antigenic Variation of the Infected Erythrocyte

**DOI:** 10.1371/journal.ppat.1000307

**Published:** 2009-02-20

**Authors:** Makhtar Niang, Xue Yan Yam, Peter Rainer Preiser

**Affiliations:** 1 Nanyang Technological University, School of Biological Sciences, Singapore, Singapore; 2 CNRS UMR 5235, University Montpellier II, Montpelier, France; Royal Melbourne Hospital, Australia

## Abstract

Modifications of the *Plasmodium falciparum*–infected red blood cell (iRBC) surface have been linked to parasite-associated pathology. Such modifications enable the parasite to establish long-lasting chronic infection by evading antibody mediate immune recognition and splenic clearance. With the exception of the well-demonstrated roles of *var*-encoded PfEMP1 in virulence and immune evasion, the biological significance of other variant surface antigens (*rif* and *stevor*) is largely unknown. While PfEMP1 and RIFIN have been located on the iRBC surface, recent studies have located STEVOR at the iRBC membrane where it may be exposed on the erythrocyte surface. To investigate the role of STEVOR in more detail, we have developed antibodies against two putative STEVOR proteins and used a combination of indirect immunofluorescence assays (IFA), live IFA, flow cytometry, as well as agglutination assays, which enable us to demonstrate that STEVOR is clonally variant at the surface of schizont stage parasites. Crucially, expression of different STEVOR on the surface of the iRBC changes the antigenic property of the parasite. Taken together, our data for the first time demonstrate that STEVOR plays a role in creating antigenic diversity of schizont stage parasites, thereby adding additional complexity to the immunogenic properties of the iRBC. Furthermore, it clearly demonstrates that to obtain a complete understanding of how parasite-induced pathology is linked to variation on the surface of the iRBC, focusing the interactions of multiple multigene families needs to be considered.

## Author Summary

Malaria remains a huge global health burden, infecting hundreds of millions of people each year. Despite a significant immune response by the host, the parasite can establish long-lasting chronic infections. Parasite-induced modifications on the surface of the infected red blood cell (rbc) are linked to the ability of the parasite to evade host immunity and may contribute to pathology. In the human parasite *Plasmodium falciparum*, proteins encoded by two parasite multigene families, called *var* and *rif*, have been shown to be exposed on the surface of the rbc. How these variant gene families help the parasite to evade the immune response and contribute to disease severity is important for the development of better antimalarial treatment and intervention strategies. Here we show that a third multigene family called *stevor* codes for proteins that are expressed on the surface of the infected rbc. We show that expression of different STEVOR changes the antigenic properties of the infected rbc, indicating a potential role of STEVOR in antigenic variation. These findings provide important new insights about the complexity of immune evasion by *P. falciparum* and in the future may lead to a better understanding of how to develop an efficient malaria vaccine.

## Introduction

During its maturation, the human malaria parasite *Plasmodium falciparum* extensively modifies the surface of the infected Red Blood Cell (iRBC). These modifications have been linked to parasite associated pathology and are thought to enable the parasite to evade antibody mediated immune recognition and splenic clearance allowing the parasite to establish long lasting chronic infection [1]. The *var*, *rifin* and *stevor* multigene family coding for *Plasmodium falciparum* erythrocyte membrane protein 1 (PfEMP1), RIFIN and STEVOR respectively have been implicated in these processes and have been postulated to play a role in host-parasite interactions. PfEMP1 is expressed on the surface of iRBCs [2],[3] where it mediates the important pathogenic traits of both clonal antigenic variation and the adhesion of iRBCs to a variety of host receptors on the endothelium of the microvasculature leading to the obstruction of blood vessels and contributing to the pathology and disease severity seen with *P. falciparum*. More recently, RIFINs have been shown to be clonally variant and to be expressed on the iRBCs surface [4],[5], although whether they are involved in pathogenesis and antigenic variation has not yet been established. Moreover, antibodies in hyper-immune sera from adults recognize RIFIN proteins, indicating that these are immunogenic, exposed, and induce malaria-specific IgG antibodies [6],[7]. Based on the high similarity in size and structure between RIFIN and STEVOR proteins and their expression patterns in parasite blood stages, these proteins are speculated to be involved in antigenic variation [8],[9].

*stevor* genes co-localized at the subtelomeric ends of the parasite's chromosomes along with the *var* and *rif* multigene families [10],[11]. Transcription of *var* initiates early after red blood cell (rbc) invasion followed by transcription of *rifin* and finally *stevor* [12],[13]. Transcription of *stevor* in single parasite seems to be restricted to a small number of genes (12), and there appears to be no coregulation of transcription between the *stevor* and *var* gene families [14]. *Stevor* and *rifin* show a similar two exon gene structure with a short exon I encoding a signal peptide while the larger exon II codes for a polypeptide possessing two predicted transmembrane domains flanking a hypervariable region [1],[15],[16]. This structure of two transmembrane domains flanking a hypervariable region appears to be conserved among a number of gene families in *P. falciparum* [8] and it has been proposed that the hypervariable region is exposed to the host immune system at some stages during the infection.

Transport of parasite proteins to the surface of iRBC occurs via Maurer's clefts (MC) [17],[18]. These organelles are believed to be important in the assembly and transport of the cyto-adherence complex of which PfEMP1 is a component to the RBC surface [5], [19]–[21]. While PfEMP1, RIFIN and STEVOR all can be detected in MC at some stage during parasite development in the RBC [12] only PfEMP1 and RIFIN have been unambiguously shown to be located on the surface of the iRBC [4],[5],[22],[23]. A recent study has shown that epitope tagged STEVOR is exported to the erythrocyte membrane [8] and this was subsequently confirmed using STEVOR specific antibodies in both lab adapted parasite lines as well as parasites directly obtained from patients [24],[25], supporting the hypothesis that it may be exposed on the erythrocyte surface and thus subjected to host immune pressure and possibly play a role in antigenic variation. Consistent with STEVOR playing a role in immune evasion is the observation that the protein is not required for parasite survival in culture and importantly that >90% of iRBC from recently isolated patient samples express STEVOR compared to <10% in long term culture adapted parasites [25].

Despite these extensive efforts there is to date no data that unambiguously demonstrates that STEVOR is indeed exposed on the surface of the iRBC and thereby contributes to antigenic variation. Importantly no experimental evidence on the validity of the current two-transmembrane domain model is currently available leaving a significant gap in our understanding on how STEVOR mediates its function. In addition the fact that only a small proportion of the population of culture adapted parasites expresses STEVOR makes it difficult to correlate transcriptional data obtained from this culture to actual protein expression and highlights the importance of linking transcription and expression data effectively. To address these important questions in more detail, we established cloned parasite lines that show uniform expression of STEVOR and developed antibodies against two putative STEVOR proteins. Using a combination of indirect immunofluorescence assays (IFA), live IFA, flow cytometry analysis as well as agglutination assays we are able to demonstrate that STEVOR is clonally variant at the surface of schizont stage parasites. Moreover, variation in the expression of STEVOR leads to changes in the immunogenic properties of the surface of the iRBC. The data obtained here challenges the currently predicted two-transmembrane domain structure of STEVOR. Taken together, our data for the first time demonstrates that STEVOR plays a role in creating antigenic diversity of schizont stage parasites, thereby adding additional complexity to the immunogenic properties of the iRBC.

## Results

### Variant expression of STEVOR in cultured parasites

**Figure 1:**
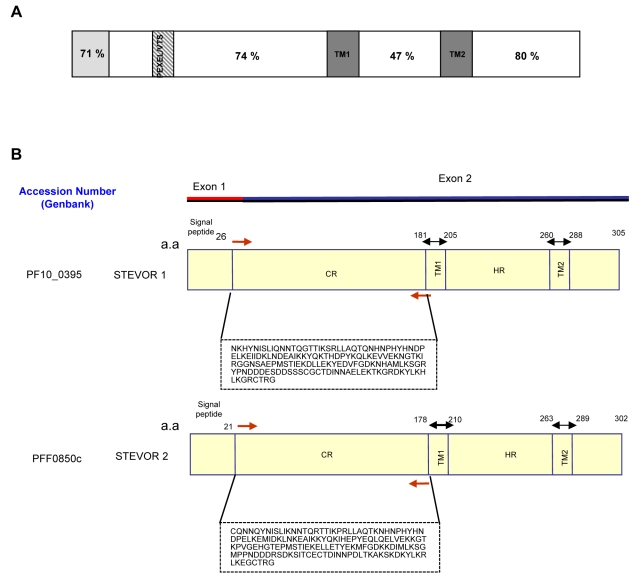
Schematic representation of STEVOR protein. (A) N-terminal signal sequences are shown in light grey, including a canonical signal sequence (encoded by exon 1) and *Plasmodium* Exported Element/Vacuolar Transport Signal (PEXEL/VTS) sites. Predicted transmembrane domains are shown in dark grey (TM1/TM2). Percentage amino acid similarity between the different regions of all available STEVOR sequences is taken from a review by Kyes et al., [13]. (B) Schematic structure of *stevor* genes with intron spliced out. Location of signal peptide, predicted transmembrane domains (TM1/TM2), conserved region (CR), and hypervariable region (HR) are indicated by amino acid (a.a) number. Orange arrows indicate primers designed to clone the CR into pET41a (+) vector for generation of *stevor* antibodies.

**Figure 2:**
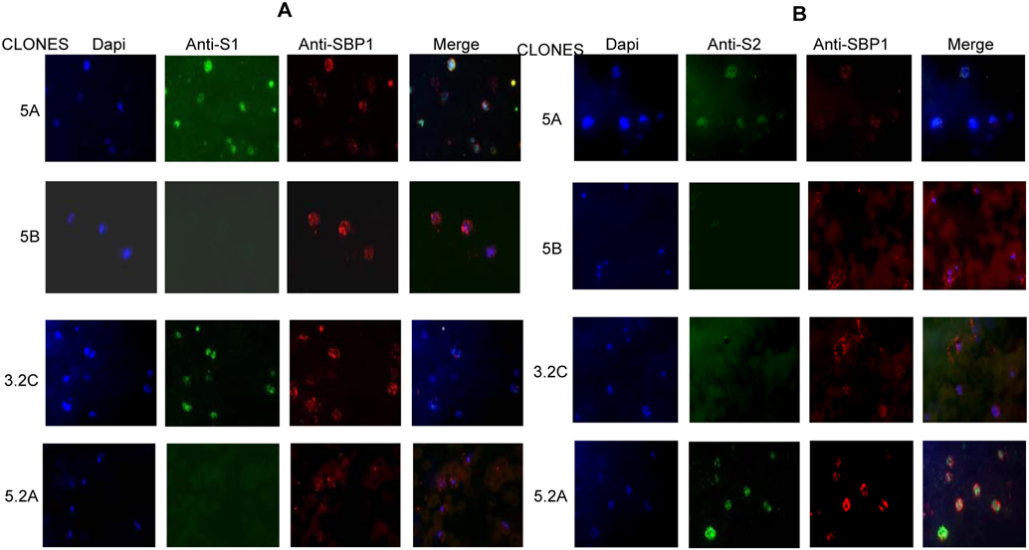
Selection of 3D7 clonal lines. Clonal lines from *P. falciparum* 3D7 parasites were isolated by limiting dilution as described in Materials and Methods. Whole field immunofluorescences co-staining of anti-PfSBP1 with either anti-S1 (A) or anti-S2 (B) antisera are shown for the four selected clones. Individual staining (dapi, anti-S1/S2, anti-SBP1) as well as merge images are shown for each clone.

Previous work had shown that while multiple *stevor* genes are transcribed in long term cultured 3D7 parasites [12], in contrast individual subclones obtained from the same population each transcribed only one or a small number of *stevor* genes [9]. In contrast, immunofluorescence microscopy using anti-STEVOR antibodies clearly showed that only a small fraction of the culture adapted parasites actually expressed STEVOR as compared to parasites derived from patients where most parasites showed STEVOR expression [25]. To establish whether it is possible to obtain cloned parasites from 3D7 parasites that stably express a single STEVOR variant; we first developed antibodies against the semi-conserved N-terminal region of STEVOR (Figure 1A). Two polyclonal sera S1 and S2 raised against two different STEVOR (Figure 1B) were used to determine the expression levels of STEVOR in four clones obtained by limiting dilution from 3D7 parasite culture (Figure 2). IFA of late stage parasites show that clone 5A is recognized by both sera S1 and S2, while clones 3.2C and 5.2A are recognized by anti-S1 and anti-S2 sera respectively (Figure 2). Clone 5B is not recognized by either S1 or S2 antisera (Figure 2) indicating that this parasite clone either does not express STEVOR or expresses a variant that is not recognized by the two anti-sera available.

**Figure 3:**
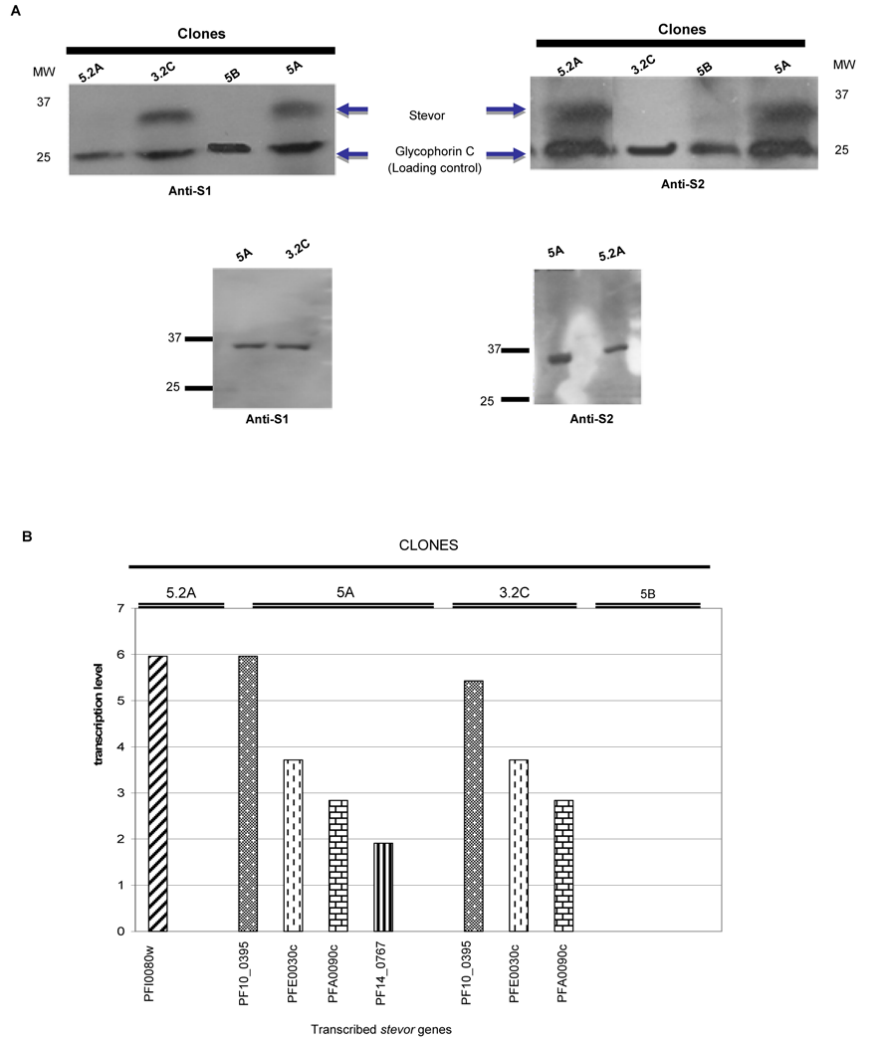
Transcription and expression analyses of STEVOR. (A) Immunoblotting of carbonate-insoluble pellet integral membrane proteins of schizont extracts of clones 5A, 5B, 3.2C, and 5.2A. A protein band of approximately 35 kDa was detected by anti-S1 serum in clones 5A and 3.2C (upper left panel) and by anti-S2 serum in clones 5A and 5.2A (upper right panel). Mouse anti-glycophorin C antibody recognizing a protein band of 25 kDa was used as loading control. Molecular weight comparison of detected STEVOR proteins by anti-S1 (lower left panel) and anti-S2 (lower right panel) is also shown. Secondary anti-rabbit (or mouse) IgG horseradish peroxydase-conjugated antibodies (Bio-Rad) and West Pico chemiluminescence (Pierce) were used to visualize the detected proteins in photographic film. Molecular marker (MM, Biorad) used for protein size comparison is shown. (B) Microarray analysis of the expression profile of transcribed *stevor* genes at mid-trophozoite stage (22–28 hours post invasion) of each clone. Level of transcription was determined in relation to a pool of RNA as described in Materials and Methods. Individual *stevor* genes with regulated transcription are shown by their accession number.

Western blot of schizonts extracts prepared from the different parasite clones confirmed the results obtained by IFA, with proteins of the appropriate size (30–40 kDa) being detected by anti-S1 in clones 5A and 3.2C (Figure 3A, upper left panel) and by anti-S2 in clones 5A and 5.2A (Figure 3A, upper right panel). No protein could be detected by either antisera in clone 5B confirming the IFA results that this parasite does not appear to express STEVOR (Figure 3A). Anti-glycophorin C serum recognizing a red blood cell membrane protein band of 25 kDa was used as loading control. Recent work [25] has shown that after sequential protein extraction STEVOR is only detected in the membrane fraction in line with being an integral-membrane protein. Consistent with this, both anti-S1 and S2 antibodies only detected the protein in the pellet fraction of the *P. falciparum* parasite clones while no STEVOR protein were detected in the supernatant and the carbonate-associated fractions (data not shown). Careful comparison of the apparent molecular weight of the STEVOR proteins detected by anti-S1 and anti-S2 indicated that while anti-S1 detected a protein band of the apparent same size in clones 5A and 3.2C there was a slight size difference in the protein recognized in 5A and 5.2A (Figure 3A, lower panel) by anti-S2. This would indicate that clones 5A and 5.2A express a different STEVOR variant that is recognized by S2. This is in line with the previous transcription studies indicating that cloned parasites stably transcribe different *stevor* [9].

To establish whether the observed differences in STEVOR expression are indeed linked to transcriptional changes in the different parasite clones, microarray analysis was performed on each clone. In line with previous studies [12],[25], peak *stevor* transcription was observed at 22–28 hours post invasion in clones 5.2A, 5A and 3.2C, with no *stevor* transcript being detected in clone 5B (Figure 3B and Table S1). Different *stevor* transcripts were detected in the different clones with one *stevor* gene detected in clone 5.2A (PFI0080w), three *stevor* (PF10_0395, PFA0090c, and PFE0030c) detected in clone 3.2C and four *stevor* (PF10_0395, PFA0090c, PFE0030c, and PF14_0767) being detected in clone 5A (Figure 3B). Previous studies using quantitative real time PCR had obtained similar results using cloned parasites with one to a few *stevor* transcript being detected in each cloned population [9]. The findings here are consistent with the western blot data where clone 5.2A is only recognized by anti-S2, clone 3.2C only being recognized by anti-S1 and clone 5A being recognized both by anti-S1 and –S2. Combining the transcriptional and western blot data it is highly likely that the STEVOR recognized by anti-S1 in clones 5A and 3.2C is PF10_0395 and PFA0090c, while PFI0080w is recognized by anti-S2 in clone 5.2A. The anti-S2 reactivity observed in clone 5A is most likely attributable to PF14_0767, while PFE0030c is considered as pseudo-gene and would not be expected to result in a functional protein.

To further validate the microarray results we further performed quantitative RT-PCR analysis targeting the genes identified by the microarray analysis in the four clones as well as PFF0850c (STEVOR 2 (Figure 1B)). Using the previously validated primers (Table S2) for the different *stevor* genes [9],[14] as well as *seryl-tRNA synthetase* (PF07_0073) as control gene [26] the transcription profile of *stevor* was analyzed at mid-trophozoite stage (22–28 hours post invasion) corresponding to the peak of total *stevor* transcript levels [12]. The findings (Figure S1) support the microarray data with the same transcription profile being detected in 5A and 3.2C clones. One additional *stevor* transcript (PF14_0767 and PFF0850c) was detected in the 5B and 5.2A clones respectively (Figure S1). Taken together, these findings support the western blot and the microarray analysis and are fully in line with previous transcription studies [9],[14] that have shown transcription of subset of *stevor* in cloned 3D7 derived parasites.

### STEVOR is located at the iRBC surface

In a recent study it has been shown that STEVOR does appear to be trafficked beyond Maurer's cleft and associate with the red blood cell membrane [8],[25]. A similar staining pattern is observed when carrying out indirect immunofluorescence assay (IFA) using anti-S1 and anti-S2 antisera and different stages of fixed iRBCs of clones 5A, 3.2C and 5.2A shown to express STEVOR (Figure S2).

**Figure 4:**
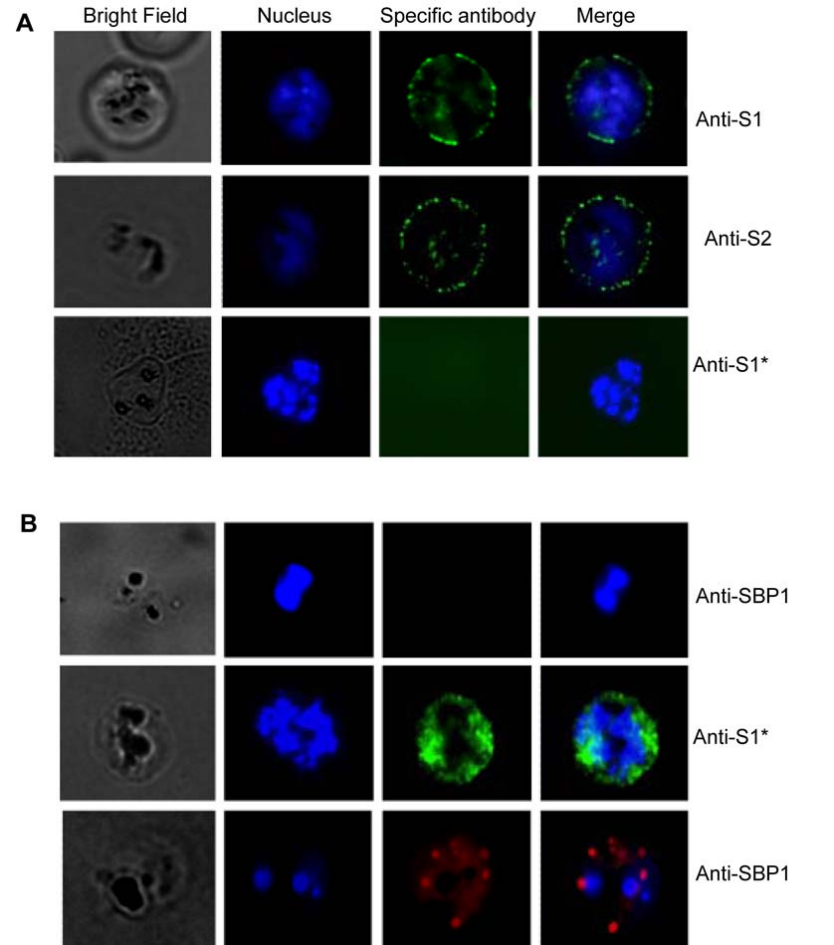
Location of native STEVOR at the iRBC surface. (A) Immunofluorescence assay of live 5A-infected erythrocytes with anti-S1 (upper row) and anti-S2 (middle row) antisera recognizing native STEVOR at the iRBC surface. Either anti-S1 or anti-S2 (lower row) did not react with trypsinized iRBC. The specific antibodies that reacted with the iRBC surface were detected with Alexa Fluor–labeled goat anti-rabbit IgG. Successive columns from left to right denote bright field, parasite nucleus staining, specific antibody staining (shown in the right), and merge images. (B) Immunofluorescence assay of live 5A-infected erythrocytes with anti-SBP1 (upper row) showing lack of staining of the iRBC. Middle and lower rows showed, respectively, anti-S1 (or anti-S2) and anti-SBP1 staining after SLO-permeabilization of the infected RBC. The specific antibodies that reacted with the iRBC surface were detected with Alexa Fluor–labeled goat anti-rabbit (or anti-mouse) IgG. The fluorescent images (individual stains and merged) and the bright-field are shown. * denotes that the same staining pattern was observed with anti-S2 antibody.

**Figure 5:**
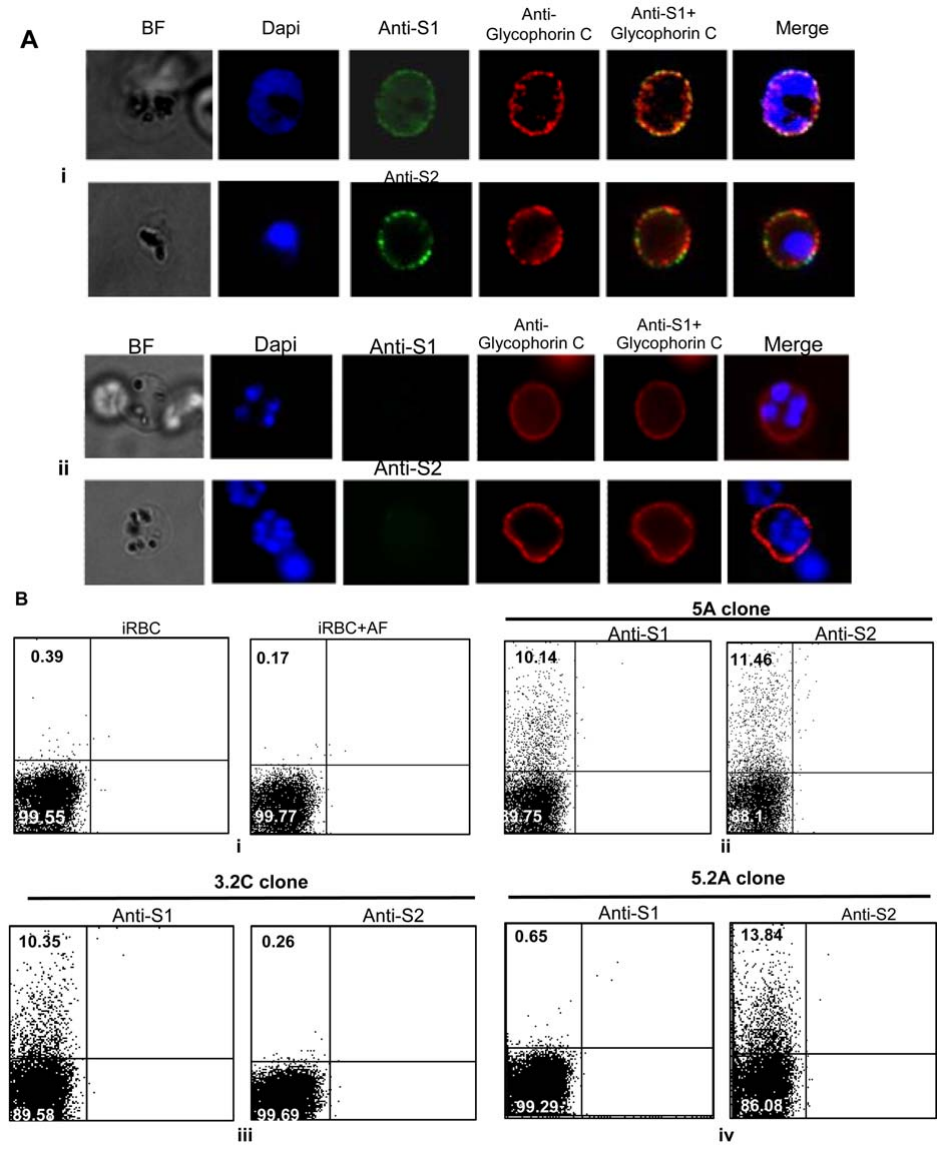
Visualization of STEVOR surface antigens. (A) Double staining of live 5A-iRBCs with either anti-S1 (or anti-S2) (panel i) with anti-Glycophorin C antibodies. Panel (ii) illustrates lack of surface STEVOR staining of 5B-iRBCs with both anti-S1 (upper row) and –S2 (lower row). The specific antibodies that reacted with the iRBC surface were detected with Alexa Fluor–labeled goat anti-rabbit (or anti-mouse) IgG. The fluorescent images (individual stains and merged) and the bright-field are shown. (B) Flow cytometry assay to measure STEVOR antigens at the iRBC surface. IRBCs alone or incubated with Alexa Fluor (AF) secondary antibody were used for controls (panel i). Live parasites of clones 5A (panel ii), 3.2C (panel iii), and 5.2A (panel iv) were incubated with anti-S1 and anti-S2 antibodies followed by the Alexa Fluor secondary antibody. Percentage of STEVOR-expressed iRBCs is shown as mean fluorescence in the upper left quadrant of each scatter plot, and Ethidium bromide–stained iRBCs appear in the lower left quadrant.

The association of STEVOR with the iRBC membrane [8],[25] raised the question on whether STEVOR is exposed on the surface. To address this, immunofluorescence staining of live parasites was carried out using anti-S1 and -S2 antibodies and the different STEVOR expressing clones. Both anti-S1 and anti-S2 antisera are able to stain the surface of live parasites (Figure 4A, upper and middle rows and data not shown) with no staining detected within the iRBC. This clearly contrasted to the IFA results obtained with fixed parasites where staining could be observed both in the MCs as well as the iRBC membrane (Figure S2) and is consistent with surface location of STEVOR. Pre-treatment of the iRBC with trypsin completely abolished the labeling of the iRBC surface in life IFAs (Figure 4A, lower row and data not shown) further supporting the surface location of STEVOR. The MC specific antibody PfSBP1 did not stain iRBC either in trypsin treated (data not shown) or untreated (Figure 4B, upper row) samples. In contrast PfSBP1 stained iRBC efficiently in this assay if the cells were permeabilized with Streptolysin O (SLO) (Figure 4B, lower row) and similarly significant staining of internal STEVOR with anti-S1 (or –S2) serum was observed following SLO-permeabilization of the iRBC (Figure 4B, middle row). Crucially, surface staining with anti-S1 and anti-S2 was only observed in parasites clones that had previously been shown to be recognized by these antibodies in IFA and Western blot analysis. Surface location of STEVOR was further supported by life IFA using antibody against the RBC membrane protein glycophorin C. Life IFA co-staining of anti-S1 (Figure 5A (i), upper row) or anti-S2 (Figure 5A (i), lower row) with anti-glycophorin C clearly showed the external location of both STEVOR and anti-glycophorin C. No STEVOR staining could be detected in life IFA with 5B-iRBCs using both anti-S1 and S2 antisera (Figure 5A (ii)).

To further confirm surface expression of STEVOR on the iRBC, flow cytometry analysis (FACS) was performed with either anti-S1 or S2 serum (Figure 5B). This clearly showed that STEVOR is detected on the surface of the iRBC of the different clones. Infected RBC of clones 5A and 3.2C were detected by anti-S1 (Figure 5B, (ii) and (iii)) in this assay while clones 5A and 5.2A reacted with anti-S2 (Figure 5B, (ii) and (iv)). No STEVOR expression could be detected with either antisera in clone 5B (Figure S3), nor did the iRBC alone or associated with the Alexa Fluor Goat anti-rabbit secondary antibody alone, acting as a negative control, stain the iRBC surface (Figure 5B, (i)). No staining of iRBC was observed with the antibody targeting PfSBP1, indicating that the observed STEVOR signal was not due to leakiness of the iRBC (Figure S3). Interestingly, permeabilization of the iRBC with SLO on the other hand now enabled PfSBP1 to penetrate the iRBC and stain the cells in the FACS analysis (Figure S3).

### Late-stage parasites iRBC show increased STEVOR surface expression

Previous studies have demonstrated that both PfEMP1 and RIFIN appear at the surface of the iRBC during the trophozoite stage [5],[27], to assess the timing of STEVOR surface expression FACS analysis using both anti-STEVOR sera was carried out on different stages of parasite development using synchronous 5A clone (Figure S3). No STEVOR expression could be detected in ring stage parasites with some expression appearing during the mid-trophozoite stage before peaking in late schizonts (Figure S3). These findings are consistent with STEVOR gradually appearing on the iRBC surface during late trophozoite and schizonts stage.

### STEVOR-specific agglutination of iRBCs

**Figure 6:**
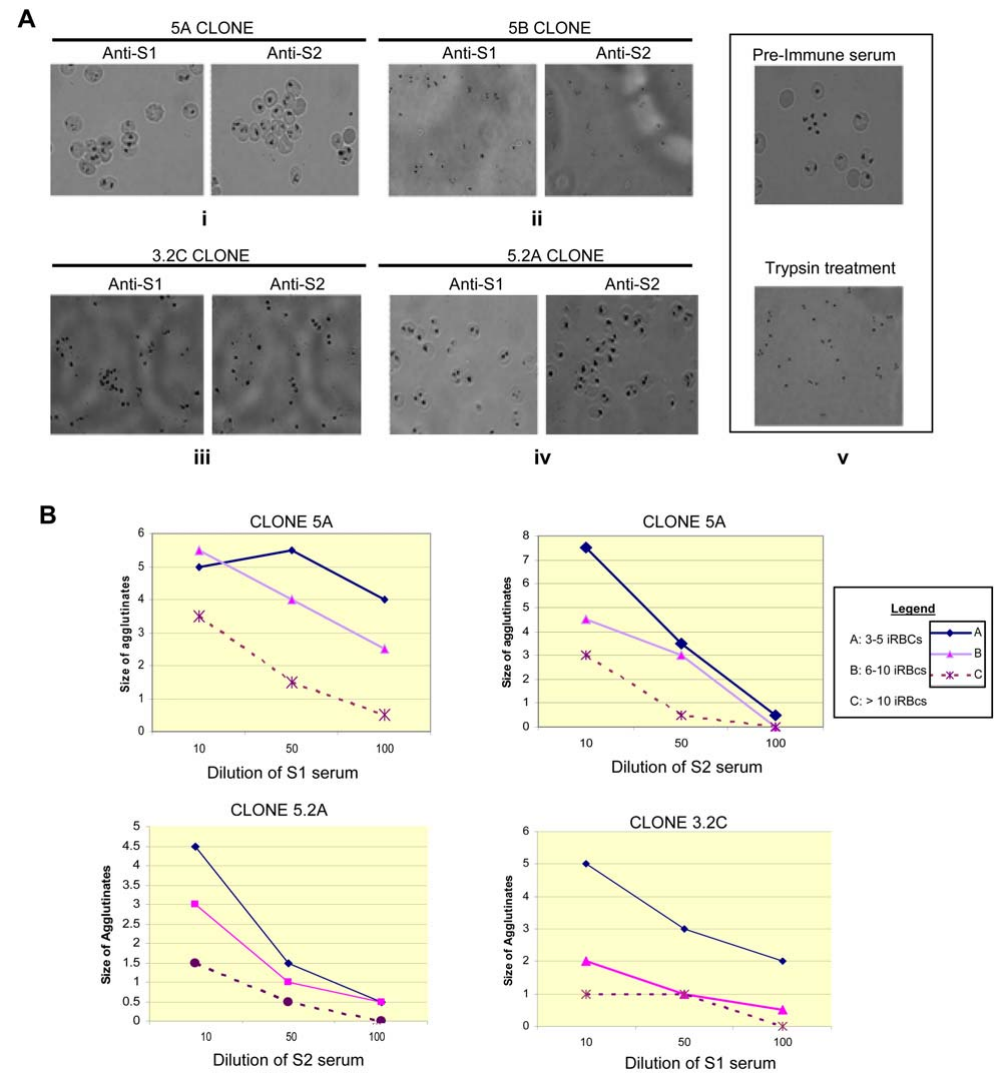
Parasites' phenotypes in agglutination assays. (A) Typical positive agglutinates of 5A-infected erythrocytes with anti-S1 and anti-S2 (panel i) sera and 3.2C (panel iii) and 5.2A (panel iv)–infected erythrocytes with anti-S1 and anti-S2 sera, respectively. Neither anti-S1 nor anti-S2 serum reacted with 5B (panel ii)–infected erythrocytes. Lack of reactivity was also observed with 5A-infected erythrocytes with pre-immune serum (panel v, upper square) and with anti-S1/S2 serum after trypsin treatment of the iRBC (panel v, lower square). (B) Proportion of iRBCs in parasites agglutinated by anti-S1 and/or anti-S2 sera. Size of the largest agglutinates decrease with increasing serum dilution. A, B, and C denote agglutinates with 3–5 iRBCs, 6–10 iRBCs, and >10 iRBCs, respectively.

Agglutination assays of iRBC provide a good sign for the ability of antibodies or a particular serum to react with the surface expressed parasite antigens [28]. The specific anti-STEVOR antibodies were tested for their ability to bind and agglutinate iRBC. Consistent with all the other data, anti-S1 antibody was only able to agglutinate 5A and 3.2C (Figure 6A, (i) and (iii)) while anti-S2 antibody agglutinates 5A and 5.2A (Figure 6A, (i) and (iv)). The proportion of iRBCs in agglutinates varies across samples and agglutinates up to 20 iRBCs were observed. A few uninfected erythrocytes were observed in agglutinates with iRBCs and did not agglutinate with other uninfected erythrocytes. These uninfected erythrocytes can be considered as contaminations. Autoagglutination being defined as self formation of aggregates containing three or more infected erythrocytes could be ruled out in our assay as either pre-immune sera (Figure 6A, (v)), or 5B parasites (Figure 6A, (ii)) did not show any agglutinates. Importantly, the *P. falciparum* strain A4, previously shown to express RIFIN but not STEVOR [5],[25] did not react with the two antibodies (Figure S4). Trypsin pre-treatment of the iRBC completely abolished the agglutination phenotype (Figure 6A, (v)) further supporting the surface location of STEVOR specific epitopes on the surface of the iRBC.

The percentage of cells in agglutinates and the size of the agglutinates (Figure 6B) decreased with successive serum dilutions indicating that the agglutination pattern reflects amount of exposed antigens at the infected erythrocytes surface. Agglutinates size and percentage of cells in agglutinates seen with both anti-S1 and –S2 in the parental 3D7 parasite were lower (Figure S4) compared to those observed in 5A clonal line (agglutinated by both S1 and S2 sera (Figure 6A, (i)), in 3.2C (Figure 6A, (iii)) and 5.2A (Figure 6A, (iv)) clones agglutinated by S1 and S2 sera respectively. These observations confirmed that agglutinating antibodies measured in the assays are clonally variant-specific and consistent with data obtained by western blot and immunofluorescence assay regarding the STEVOR expression of the different clones.

## Discussion

The expression of variant antigens on the surface of *P. falciparum* infected red blood cells has been linked to immune evasion and pathology. While the *var*, *rifin* and *stevor* multigene families are the largest gene families so far identified in *P. falciparum*, our understanding of the role they play in parasite host interaction is limited. Only for PfEMP1 has a functional role in adhesion to the host endothelium via host specific receptors, the binding of uninfected rbc to form rosettes, as well as antigenic variation been demonstrated [16],[22],[29],[30]. RIFIN are known to be located on the surface of iRBC [5] and there is evidence that different RIFIN are expressed in different parasite lines, leading to the postulation that RIFIN play an additional role in antigenic variation [5],[8]. In addition RIFIN have also been proposed to play a role in rosette formation though this needs to be confirmed. Recently, an additional role in merozoite invasion or immune evasion has been proposed for RIFIN based on the expression of a subset of RIFIN in merozoites [31].

The role of STEVOR is even less clear. Its timing of expression after both PfEMP1 and RIFIN indicated that it has a different function [12] and this was supported by the initial observations that unlike RIFIN and PfEMP1 the protein did not get trafficked to the iRBC surface but remained inside MC [12],[32]. Recent studies showing that STEVOR moves out of the MC in late stage parasite [8],[24],[25] and associates with rbc membrane have challenged this idea. These studies though were unable to unequivocally demonstrate that STEVOR is indeed presented at the surface of the iRBC thereby being directly exposed to the host immune system. Here for the first time we directly demonstrate that STEVOR is expressed on the surface of the iRBC. We show that STEVOR moves out of MC in late stage parasite and is moved to the RBC surface where it is recognized by STEVOR specific antibodies. The appearance of STEVOR on the surface is after both PfEMP1 and RIFIN and would indicate that STEVOR plays an important and distinct role in the later stage of the parasite development in the RBC.

The extensive sequence diversity of different members of the STEVOR family is in line with the protein being under selective pressure by the immune system and our findings that the protein is surface expressed is consistent with this. Interestingly, our data contradict the predicted structure of STEVOR (Figure 1A) where two transmembrane (TM) domains flank a surface exposed short hypervariable region [10],[15]. Rather our data clearly show that the more semiconserved N-terminal region is exposed on the surface. Based on the current model this would mean that the hypervariable region is located on the inside of the RBC with the N- and C-terminal parts of the protein being exposed. Our alternative model is that the TM domain predicted between the N-terminal and hypervariable region is not used and both regions are therefore exposed on the surface of the iRBC being anchored to the RBC membrane by the second C-terminal TM region. This needs to be explored further but it highlights the importance of validating predictions about the membrane topology of parasite proteins.

We have previously been able to show that STEVOR is not needed for survival of culture adapted parasites and this is seen by the overall low expression of this protein in different parasite lines [12],[25]. This contrasts with expression of STEVOR in parasites directly isolated from patients where transcription as well as expression levels of STEVOR are much higher [25],[33], indicating an important role in the host. In this work we have now been able to obtain parasite clones from the 3D7 strain that express different members of STEVOR at high levels. This has enabled us to unambiguously show surface location of STEVOR on the iRBC but also for the first time provide a tool for studying the antigenic diversity of STEVOR. While *var* gene switching leading to the expression of different members of PfEMP1 on the surface of the iRBC has been clearly demonstrated it has up to date been much more difficult to link this to variation in immunogenic properties of the iRBC [34]. Similarly, while antibodies recognizing RIFIN can be found in natural infections it is again not clear how changes in the RIFIN expression pattern impact on the recognition of the iRBC. In our study we for the first time have generated antibodies that can distinguish between different STEVOR proteins. These antibodies have enabled us to convincingly demonstrate that STEVOR is able to clonally vary on the surface of the iRBC and importantly that these variations change the antigenic properties of these cells. Based on these findings it is possible to conclude that STEVOR plays a role in the antigenic variation of the late developmental stages of the blood stage parasite.

Transcriptional analysis of *stevor* in clones derived from the parasite strain NF54 show stable expression of a small number of *stevor* genes over a significant period of time. How this correlated with actual protein expression was not known. In our study we are now able to show stable expression of a particular STEVOR variant in single parasite clones over more then 30 generations. It will be interesting to establish how quickly STEVOR expression changes in the presence of an active immune response. At this stage there is no evidence of a link between STEVOR antibodies and clinical outcome, the fact that here now convincing evidence showing surface expression and clonal antigenic variation of STEVOR makes this now an important question to pursue.

In summary, we clearly show that STEVOR is located on the surface of *P. falciparum* infected RBC. We furthermore show that STEVOR expression is clonally variant and that expression of different STEVOR variants changes the antigenic properties of the iRBC. This strongly supports the role of STEVOR in antigenic variation. Whether STEVOR mediates certain adhesive functions in addition to immune evasion needs now to be investigated. At this stage it is not clear why the malaria parasite needs an additional variant antigen on the surface of the iRBC and more studies are required to address this question.

## Materials and Methods

### Parasite culture

Well characterized *P. falciparum* laboratory parasites lines (3D7 and A4 clone (kind gift of Sue Kyes)) and clonal lines thereof, were continuously cultured in fresh human RBCs in RPMI 1640 completed medium supplemented with 10% albumax as previously described [35]. If required, cultures with a parasitemia of approximately 5% (mainly ring stage) were synchronized by treatment with 5% (w/v) sorbitol as described [36],[37]. Late stage-schizont iRBCs were purified by treatment with 70% Percoll as described by [38].

Tighter synchronization (Figure S3) was achieved by adding purified schizont stage iRBC to fresh RBC for reinvasion. After four hours the culture was treated with sorbitol to remove mature stage parasites as previously described (38).

### Bioinformatics

Sequence data, gene structure, predicted domains and protein sequence for all *stevor* genes were obtained from PlasmodDB (http://www.plasmodb.org/). NCBI BLAST (http://www.ncbi.nlm.nih.gov/BLAST/) and ClustalW were used to check for sequence alignment.

### Generation of Stevor 1 and Stevor 2 antibodies

Two putative *stevor* genes (accession numbers: PF10_0395 and PFF0850c) were used for generation of stevor antibodies. Generation of stevor 1 antibody has been previously described [25] and the same approach was used for generation of stevor 2 antibody. Schematic structure of the genes showing the location of the predicted signal sequence, transmembrane domains, semi-conserved regions and hypervariable regions is represented (Figure 1A and 1B).

Primers (Figure 1B) to conserved region were designed to amplify the DNA sequence and the PCR products were cloned into the pET-24a (+) vector (Novagen) and expressed in BL21-RIL strain (Stratagene, California, USA). The fusion proteins were purified using two-step purification method via Nickel colums (Qiagen) according to manufacturer's protocol.

Recombinant proteins were used for immunization of rabbits and generation of polyclonal stevor antibodies. These latest have been renamed as S1 and S2 antisera which were used for all subsequent experiments.

### Generation of *P. falciparum* clonal lines

Clonal lines of *P. falciparum* 3D7 parasites were isolated by limiting dilution using 96-well microtitre plates as previously described [39]. Positive wells were identified by Giemsa staining over a period of 14 to 21 days after cloning. Different clones were screened by immunofluorescence assay co-staining with anti-PfSBP1 and either anti-S1 (Figure 2A) or –S2 (Figure 2B) sera. Four clonal lines (5A, 5B, 3.2C and 5.2A) showing different staining patterns against anti-S1 and –S2 (Figure 2A and 2B) sera were further selected for subsequent experiments.

### Immunofluorescence assays (IFA) of fixed parasite preparations

Thin blood smears were prepared at different stages of highly synchronized *P. falciparum* parasites through the erythrocytic life cycle. Slides were fixed at 4°C in acetone for 5 min, wrapped in foil and stored at −20°C. When required, slides were allowed to reach room temperature, blocked with Diluent Buffer 2 (PBS/1%BSA/0.05% sodium azide) and then incubated for 1 h in a humid chamber with primary following antibodies: rabbit anti-S1 (1∶200) and –S2 (1∶400) antibodies and mouse (B28) anti-*P. falciparum* skeleton binding protein 1 (anti-PfSBP1) (1∶200) (kind gift of Catherine Braun-Breton). This was followed by secondary antibodies: Alexa Fluor 488 coupled anti-rabbit (1∶200) and Alexa Fluor 594 coupled anti-mouse (1∶200) antibodies (Molecular Probes). Slides were then briefly dipped three times in 4,6-diaminido-2-phenylindole (DAPI, 2 µg/mL in PBS) for parasite nuclei staining and viewed under Olympus fluorescent microscope.

### Western blotting

Schizonts of 44–48 hours from different cultures at 5% parasitemia were enriched by Percoll as previously described [38]. Schizont extracts or uninfected RBCs were fractionated into three protein populations: soluble proteins (S), carbonate-peripherally membrane-associated proteins (C), and pellet-integral-membrane proteins (P) by sequential protein extraction as previously described [40]. Different fractions were then separated by Sodium dodecyl sulfate-polyacrylamide gel electrophoresis (SDS-PAGE) and transferred onto nitrocellulose membrane. To detect STEVOR proteins, membrane were probed with rabbit anti-S1 and anti-S2 polyclonal sera diluted in PBS/0.05%Tween at 1∶5000 and 1∶1000 respectively, followed by the respective horseradish peroxidase (HRP)-conjugated secondary antibody enhanced by SuperSignal West Pico Chemiluminescent Substrate (PIERCE) according to manufacturer's recommendations. Anti-glycophorin C (Ret40f) antibody purchased from V-Cell Science (Santa Cruz Biotechnology) was used as loading control to ensure that same amount of protein from membrane fraction was loaded in each lane.

### Agglutination assay

*P. falciparum* trophozoites-iRBCs (∼5% parasitemia) were synchronized by purification by Percoll gradient as previously described [38]. Fresh uninfected erythrocytes were added to ensure that the parasitemia of pigmented trophozoites was around 5%. Cells were diluted to 5% heamatocrit in PBS containing ethidium bromide (10 µg/ml) (Sigma). 22.5 µl of parasites suspension were added to 2.5 µl either anti-S1 or -S2 serum (final dilution 1/10) or pre-immune serum in 1.5 ml Eppendorf tubes. Samples were incubated at room temperature for 45 min on a rotating wheel (30 rpm). Following gentle resuspension, 10 µl of cells suspension was gently smeared in a circular motion with a plastic micropipette tip onto glass slides as previously described [41]. Duplicate 1.5 cm diameter smears were made, air-dried for 5 min, fixed in methanol and stained for 10 min with 10% Giemsa's stain. Smears were then preserved with mounting medium and a glass cover slip. Samples were examinated by light microscopy and classified as positive by agglutination if they contain ≥3 agglutinates of intact iRBCs. Size of agglutinates were quantified as follows: size A, 3 to 5 cells; size B, 6 to 10 cells; size C, >10 cells.

### Live immunofluorescence assay and flow cytometry analysis of the iRBC surface

Live immunofluorescence assay (Protocol S1) was performed on parasites when the majority were ideally in late schizont stage. IRBCs were pelleted from culture by centrifugation at 800×g for 3 minutes, washed thrice with RPMI 1640 w/o albumax. Parasitemia was diluted to 5% with uninfected erythrocytes and parasites were resuspended at 5% heamatocrit. Fifty µl of cells suspension containing either anti-S1, -S2 rabbit sera or ant-SBP1 mouse serum at different dilutions (1∶10–1∶400) were incubated for 1 h at room temperature, following which the cells were re-suspended in 50 µl of secondary anti-mouse or rabbit IgG antibody at the appropriate dilution in RPMI 1640 containing ethidium bromide (1 µg/ml). Following 1 h incubation, a drop of cells was placed onto a glass slide, mixed with 10 µl mounting medium, then cell surface staining was visualized with a Olympus fluorescence microscope at 100× magnification.

For flow cytometry analysis, RPMI 1640 was replaced by PBS containing 0.1% BSA. Cells (10.000) were counted on a FACs Calibur using Cell Quest software (Becton Dickinson, San Jose, CA, USA). Gates were set using either iRBCs only or stained with alexa fluor secondary antibody. The amount of IgG bound antigens on the erythrocyte surface was then measured as the mean fluorescence signal. The different parasites staining were visualized in dot plots as fluorescence in FL1 channel.

### Permeabilization of iRBCs with pore-forming Streptolysin O

Streptolysin O (SLO) was purchased from Sigma Chemical Co (St. Louis, MO, U.S.A.) and prepared by method that allows post-activation storage as previously described [42]. Briefly, SLO (25 000 units) was re-suspended in 1 ml of PBS and aliquots were stored at −20°C. Prior to use, SLO aliquots were thawed at room temperature and activated by incubation with 100 mM dithiothreitol for 15 min. For permeabilization, 2×10^8^ iRBCs were resuspended in 200 µl of buffer (1XPBS/1% BSA) containing 3–4 heamolytic units of SLO as defined previously [43] and incubated at room temperature for 6 min. Cells were pelleted at 850×g for 5 min and washed twice with PBS/1%BSA. The pellet (permeabilized iRBCs) was analyzed by live immunofluorescence and FACS analysis (as described above) for specific PfSBP1 staining.

### Trypsin treatment of *P. falciparum* cultures

Intact iRBCs were pelleted and resuspended in trypsin (Sigma Chemical) solution (1 mg/ml in RPMI 1640 w/o albumax) for 5 min at 37°C with constant rotation. The reaction was stopped by addition of trypsin inhibitor (Sigma Chemical) (1 mg/ml) for another 5 min. Cells were washed with RPMI 1640 w/o albumax or PBS and resuspended in PBS.

### Microarray analysis

RNA obtained from different time points of synchronized *P. falciparum* intracellular blood-stage parasites of 5A, 3.2C, 5.2A and 5B clones were hybridized against a reference pool from the same clone as previously described [44] using a *P. falciparum* long oligonucleotide microarray [45]. Microarray analysis, data acquisition and analysis were performed (Protocol S1) as recently described [25].

## Supporting Information

Protocol S1 Supplemental materials and methods
(0.03 MB DOC)

Table S1 Transcriptional analysis of *stevor* gene family in the different clones. Summary of microarray analysis of transcribed *stevor* genes during asexual blood stage from 24 hrs after invasion and onward in the four clones. Relative transcription levels of 34 *stevor* genes are shown. Analysis was performed as described in the Materials and Methods section. Bold P+ denotes highly transcribed genes, P- denotes low transcription, and ND denotes nondetected genes. Genes are shown by their accession numbers.
(0.06 MB XLS)

Table S2 Specific primers sets used to amplify the 6 *stevor* genes and the seryl-tRNA synthetase housekeeping gene
(0.21 MB TIF)

Figure S1 Transcriptional levels of selected *stevor* genes in the four clones. Analysis of the *stevor* gene was performed at 24–28 hours post-invasion for the four clones. Hatched grey bar represents level of transcription of the seryl-tRNA synthetase housekeeping gene.
(0.22 MB TIF)

Figure S2 Indirect immunofluorescence assay (IFA) of asexual stages of the 5A clone. Immunofluorescence staining of mature (>24 hour) blood-stage *P. falciparum* parasites using anti-S1 (A) and anti-S2 sera (B). Staining of trophozoite (T) and late schizont (LS) stage parasites are shown. Parasites' proteins were stained with anti-S1 or -S2 rabbit serum (green), the Maurer's cleft-specific anti-PfSBP1 mouse serum (red). Parasites' nuclei were stained with DAPI (2 µg/ml) (blue). The individual stains as well as the merged (S1/S2+SBP1, merge) images are shown. BF denotes bright field image.
(1.02 MB TIF)

Figure S3 Timing of STEVOR surface expression
(0.21 MB TIF)

Figure S4 Agglutination assays
(0.59 MB TIF)
