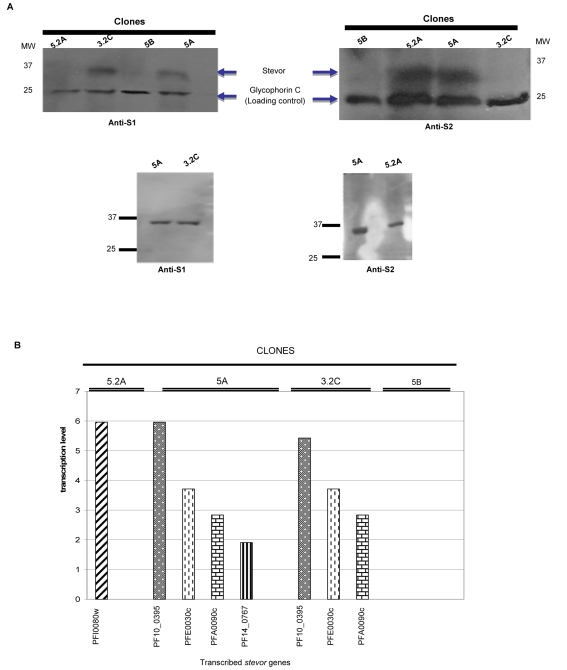# Correction: The *Plasmodium falciparum* STEVOR Multigene Family Mediates Antigenic Variation of the Infected Erythrocyte

**DOI:** 10.1371/annotation/c58250db-8cce-40c5-b7ac-42204050069a

**Published:** 2009-09-29

**Authors:** Makhtar Niang, Xue Yan Yam, Peter Rainer Preiser

In Figure 3A there was a discrepancy between the original blot and the Anti-S1 blot, as published. Please see the corrected Figure 3 here: 

**Figure:**