# Correction: The *Plasmodium falciparum* STEVOR Multigene Family Mediates Antigenic Variation of the Infected Erythrocyte

**DOI:** 10.1371/annotation/6b4264ca-dd6a-4255-9f91-c0fb6692cc26

**Published:** 2011-09-26

**Authors:** Makhtar Niang, Xue Yan Yam, Peter Rainer Preiser

This Correction is a clarification of the original Correction notice that was posted on this article concerning Figure 3. In the corrected figure, both of the upper panels in Figure 3a have been replaced (labeled Anti-S1 and Anti-S2) with new versions. The equivalent panels in the original figure comprised lanes that had been rearranged and were incorrectly labeled, including two lanes in the original right hand panel that were inadvertantly duplicated. The replacement blots are presented without any rearrangement of the lanes, and the conclusions of this experiment and the others presented in the article remain unaffected by the errors in the original figure.

