# Supplementary material for: The Plasmodium falciparum STEVOR Multigene Family Mediates Antigenic Variation of the Infected Erythrocyte
Source: PLoS Pathog. 2009 Feb 20;5(2):e1000307. doi: 10.1371/journal.ppat.1000307 (PMC2637975; doi:10.1371/journal.ppat.1000307)
Supplement: Protocol S1 — Supplemental materials and methods (0.03 MB DOC) [file ppat.1000307.s001.doc]

**Protocol S1**

**Supplemental Materials and Methods**

**Live Immunofluorescence assay**

Live immunofluorescence assay was performed as described in Material and methods. Mouse monoclonal anti-Glycophorin C (Ret40f) antibody was purchased from V-Cell Science (Santa Cruz Biotechnology) and used at 1:200 dilution followed by Alexa fluor 594 coupled anti-mouse antibody (Molecular Probes) secondary antibody diluted at 1:400. Cell surface staining was visualized with Olympus fluorescence microscope at 100X magnification.

**RNA extraction, microarray analysis and RT-PCR**

Pellet obtained from different time points of synchronized *P. falciparum* blood-stage parasites of 5A, 5B, 3.2C and 5.2A clones was used for RNA isolation. Total RNA was prepared with Trizol (Invitrogen) as recommended by the manufacturer and treated with Dnase I (Invitrogen) until free of DNA. For microarray analysis, DNA free RNA from the different time points were hybridized against a reference pool from the same clone as previously described (Bozdech *et al*., 2003) using a *P. falciparum* long oligonucleotide microarray (Hu *et al*., 2007). Microarray analysis, data acquisition and analysis were performed as recently described (Blythe *et al*., 2008).

For RT-PCR, a total of 2µg of DNA free RNA was reverse-transcribed using Superscript II and primed with random hexamers primers (Invitrogen) in 20µl volume. Reverse transcription was performed at 42°C for 50 min.

Real time PCR was carried out in triplicate using ABI Prism 7900HT sequence detector (Applied Biosystems). Each reaction was optimized to contain equal amount of cDNA, 1µM of gene specific primers and 1X SYBR Green PCR-master mix (Applied Biosystems) in 20 µl volumes.

Gene specific primers (Table S2) were obtained from published primers sets (Sharp *et al*., 2006; Lavazec *et al*., 2007). The control gene *seryl-tRNA synthetase* primers previously described by Salanti *et al.* (2003) were used as control in all reactions. The control gene was identified in the *P. falciparum* 3D7 genome sequence database ([http://www.plasmodb.org](http://www.plasmodb.org/)) as fulfilling transcript expression criteria for housekeeping control genes which display consistent transcript expression patterns throughout the parasite life cycle. The relative copy number for each gene was determined as the ratio of the relative amount of target gene cDNA/the relative amount of housekeeping control gene cDNA.
