# Supplementary figures and images for: The Plasmodium falciparum STEVOR Multigene Family Mediates Antigenic Variation of the Infected Erythrocyte
Source: PLoS Pathog. 2009 Feb 20;5(2):e1000307. doi: 10.1371/journal.ppat.1000307 (PMC2637975; doi:10.1371/journal.ppat.1000307)

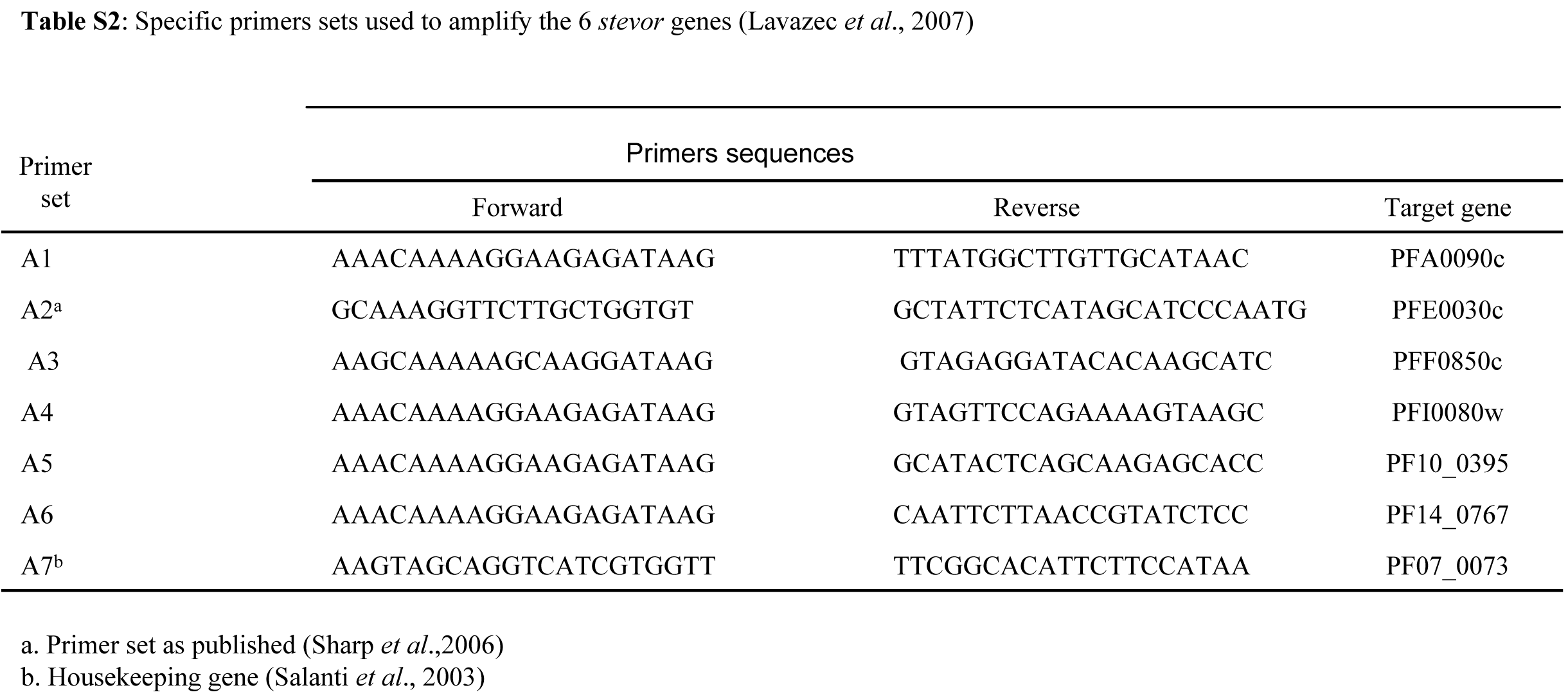

Supplement: Table S2 — Specific primers sets used to amplify the 6 stevor genes and the seryl-tRNA synthetase housekeeping gene (0.21 MB TIF) [file ppat.1000307.s003.tif]

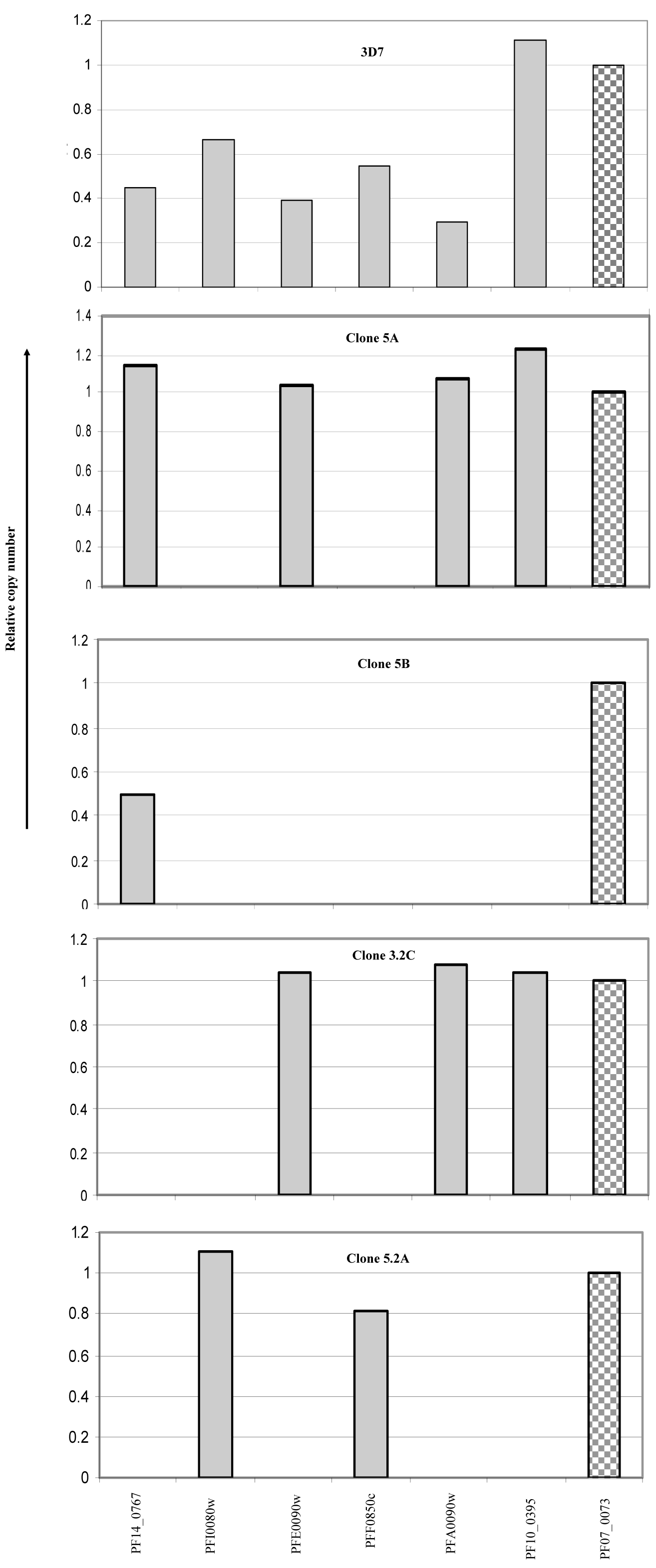

Supplement: Figure S1 — Transcriptional levels of selected stevor genes in the four clones. Analysis of the stevor gene was performed at 24–28 hours post-invasion for the four clones. Hatched grey bar represents level of transcription of the seryl-tRNA synthetase housekeeping gene. (0.22 MB TIF) [file ppat.1000307.s004.tif]

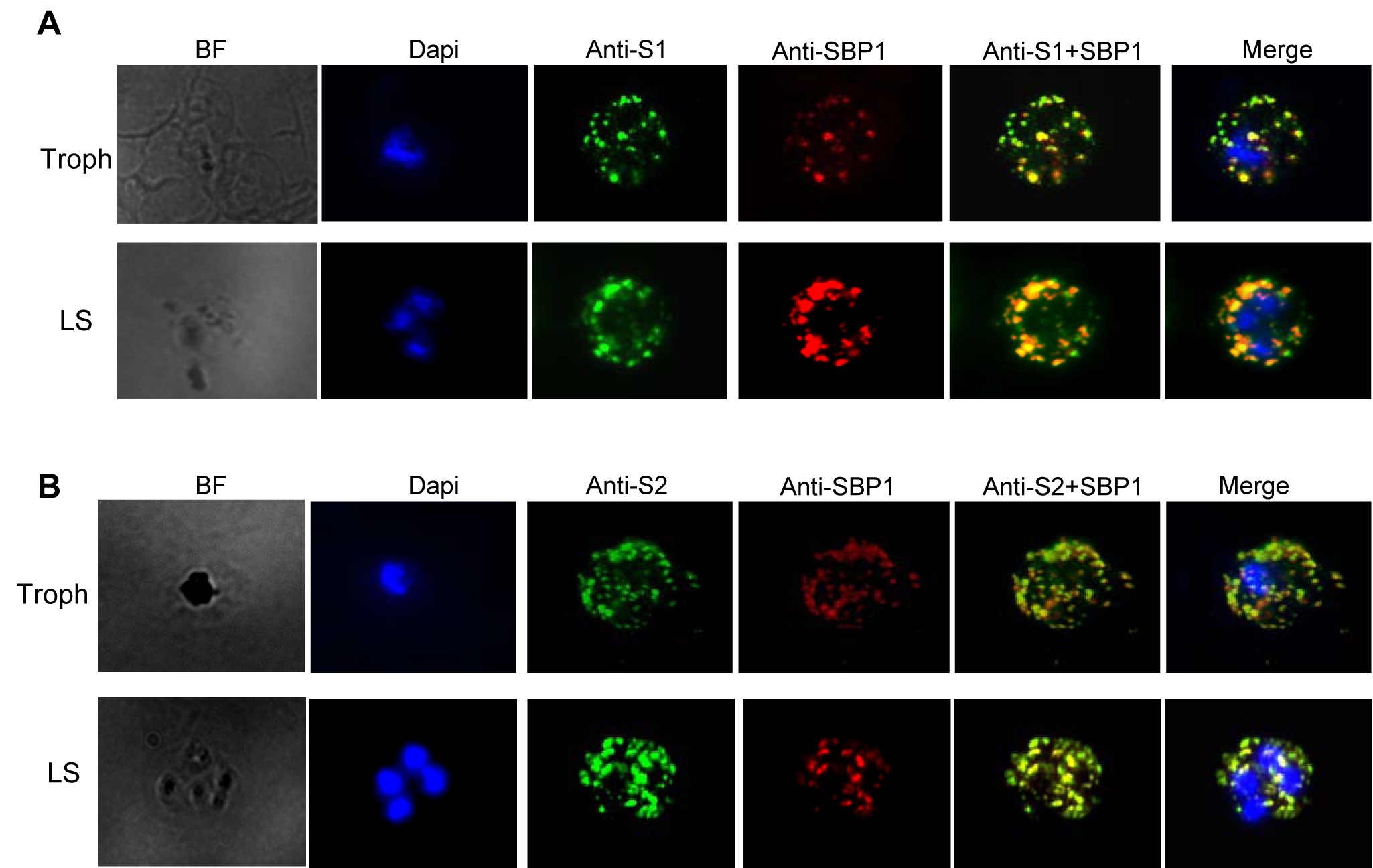

Supplement: Figure S2 — Indirect immunofluorescence assay (IFA) of asexual stages of the 5A clone. Immunofluorescence staining of mature (>24 hour) blood-stage P. falciparum parasites using anti-S1 (A) and anti-S2 sera (B). Staining of trophozoite (T) and late schizont (LS) stage parasites are shown. Parasites' proteins were stained with anti-S1 or -S2 rabbit serum (green), the Maurer's cleft-specific anti-PfSBP1 mouse serum (red). Parasites' nuclei were stained with DAPI (2 µg/ml) (blue). The individual stains as well as the merged (S1/S2+SBP1, merge) images are shown. BF denotes bright field image. (1.02 MB TIF) [file ppat.1000307.s005.tif]

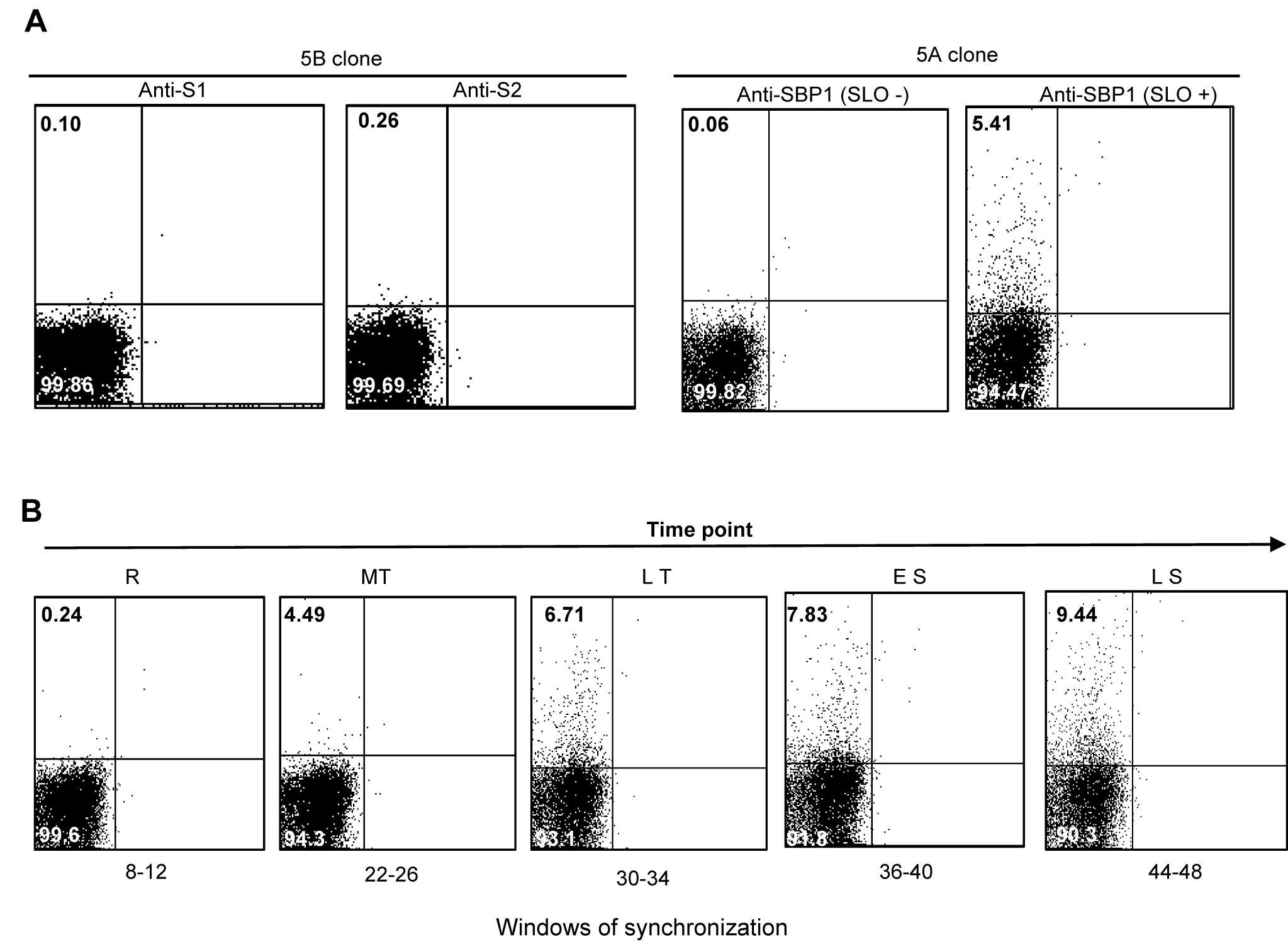

Supplement: Figure S3 — Timing of STEVOR surface expression (0.21 MB TIF) [file ppat.1000307.s006.tif]

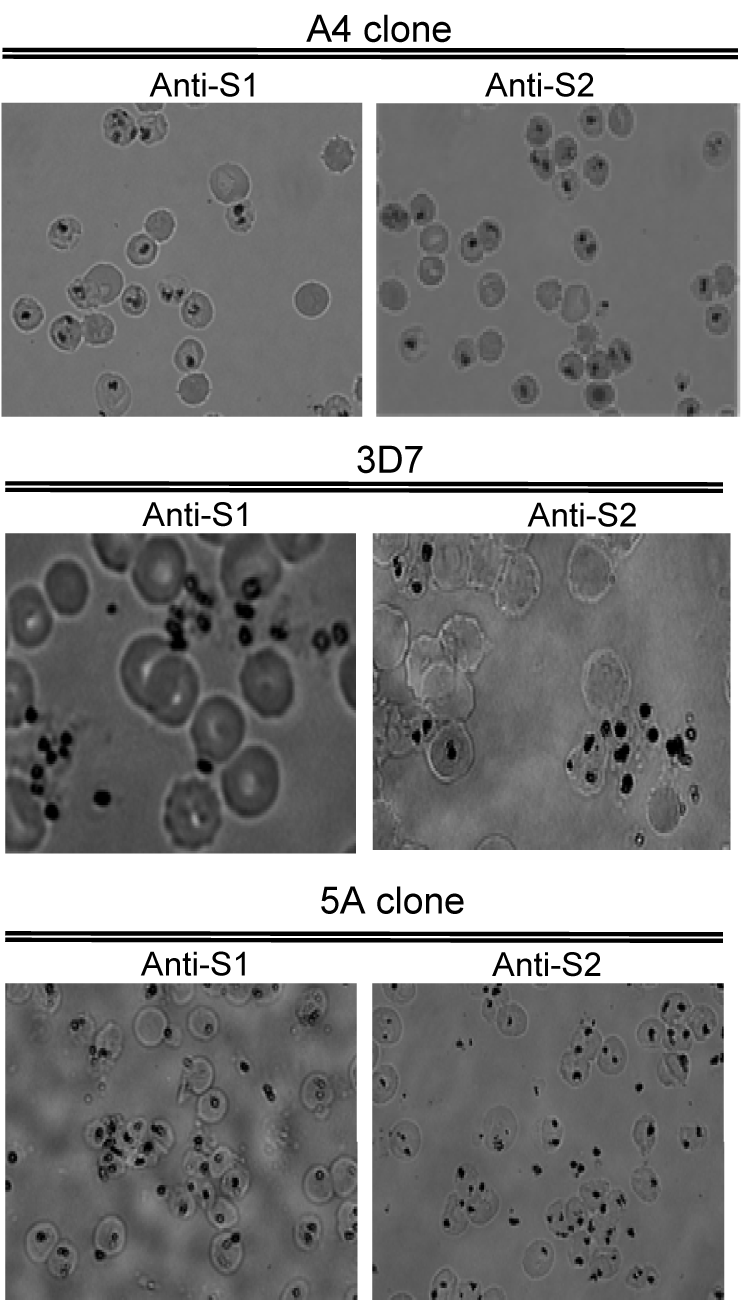

Supplement: Figure S4 — Agglutination assays (0.59 MB TIF) [file ppat.1000307.s007.tif]
